# A qualitative analysis of algorithm-based decision support usability testing for symptom management across the trajectory of cancer care: one size does not fit all

**DOI:** 10.1186/s12911-024-02466-7

**Published:** 2024-03-05

**Authors:** Hayley Dunnack Yackel, Barbara Halpenny, Janet L. Abrahm, Jennifer Ligibel, Andrea Enzinger, David F. Lobach, Mary E. Cooley

**Affiliations:** 1 Hartford HealthCare Cancer Institute, 80 Seymour Street, 06106 Hartford, CT USA; 2 Dana-Farber Cancer Institute, 450 Brookline Ave, LW-508, 02215 Boston, MA USA; 3 Elimu Informatics, 1709 Julian Court, 94530 El Cerrito, CA USA

**Keywords:** Cancer symptom management, Clinical decision support, Clinical practice guidelines, Cancer-related fatigue, Cancer-related constipation

## Abstract

**Background:**

Adults with cancer experience symptoms that change across the disease trajectory. Due to the distress and cost associated with uncontrolled symptoms, improving symptom management is an important component of quality cancer care. Clinical decision support (CDS) is a promising strategy to integrate clinical practice guideline (CPG)-based symptom management recommendations at the point of care.

**Methods:**

The objectives of this project were to develop and evaluate the usability of two symptom management algorithms (constipation and fatigue) across the trajectory of cancer care in patients with active disease treated in comprehensive or community cancer care settings to surveillance of cancer survivors in primary care practices. A modified ADAPTE process was used to develop algorithms based on national CPGs. Usability testing involved semi-structured interviews with clinicians from varied care settings, including comprehensive and community cancer centers, and primary care. The transcripts were analyzed with MAXQDA using Braun and Clarke’s thematic analysis method. A cross tabs analysis was also performed to assess the prevalence of themes and subthemes by cancer care setting.

**Results:**

A total of 17 clinicians (physicians, nurse practitioners, and physician assistants) were interviewed for usability testing. Three main themes emerged: (1) Algorithms as useful, (2) Symptom management differences, and (3) Different target end-users. The cross-tabs analysis demonstrated differences among care trajectories and settings that originated in the Symptom management differences theme. The sub-themes of “Differences between diseases” and “Differences between care trajectories” originated from participants working in a comprehensive cancer center, which tends to be disease-specific locations for patients on active treatment. Meanwhile, participants from primary care identified the sub-theme of “Differences in settings,” indicating that symptom management strategies are care setting specific.

**Conclusions:**

While CDS can help promote evidence-based symptom management, systems providing care recommendations need to be specifically developed to fit patient characteristics and clinical context. Findings suggest that one set of algorithms will not be applicable throughout the entire cancer trajectory. Unique CDS for symptom management will be needed for patients who are cancer survivors being followed in primary care settings.

**Supplementary Information:**

The online version contains supplementary material available at 10.1186/s12911-024-02466-7.

## Background

Most patients with cancer have distressing symptoms that are often undertreated and as a result increase morbidity, mortality and cost [1]. Uncontrolled cancer symptoms impose a significant burden for patients and are associated with increased emotional distress and decreased function [1, 2]. Given the negative sequelae associated with uncontrolled symptoms, improving symptom management is an important component of quality cancer care [3].

Core symptoms are defined as symptoms that are prevalent in cancer care and commonly occur across different tumor and treatment types [4]. Some of these core symptoms are co-occurring so it is necessary to assess multiple symptoms in the clinical setting [5]. Research suggests that these symptoms should be assessed by patient reported outcome (PRO) measures [4, 5]. Two prevalent core symptoms in cancer care are fatigue (reported by 62% of patients during treatment [6]) and constipation (reported by ∼ 60% of patients with cancer [7]). Efforts to assess and treat these prevalent core symptoms have important implications for clinicians caring for cancer patients.

An evidence-based approach to symptom management includes the use of clinical practice guidelines (CPGs). These CPGs are widely available and could impact practice but are not often implemented [8–11] because time constraints and inadequate assessment of symptoms pose significant barriers to their integration into clinical practice [12]. A promising strategy for CPG-integration includes the development of CDS systems for symptom management (CDS-Sx), defined as “computerized programs providing clinicians with person-specific information that is intelligently filtered and presented at the appropriate time to enhance health care” [13]. The implementation of algorithm-based CDS-Sx in the clinical setting seeks to seamlessly integrate CPGs into daily practice, representing a best practice initiative to enhance “meaningful use” of the electronic health record (EHR) to improve outcomes. In accordance with the HITECH Act of 2009, CDS-Sx optimizes communication between patients and clinicians with a unique structure that emphasizes guideline-informed care [14, 15]. A key component of algorithm-based CDS-Sx includes end-user involvement across the development phases [16].

Prior studies investigating cancer symptom management through CDS used paper, telephone, or electronic delivery methods to implement the intervention. Among the studies that used an electronic-based delivery approach, symptoms were monitored on a regular basis and an alert was used to notify clinicians if symptom severity passed a certain threshold and/or educational materials were delivered to patients to promote self- management of symptoms [17–19]. None of the electronic-based systems used an algorithm-based approach for management of cancer symptoms. The previous studies using algorithm-based CDS were paper-based and centered on management of a solitary symptom [20–22]. These prior studies demonstrated that after algorithm implementation, there was increased adherence to symptom management guidelines and improved patient outcomes for individual symptoms (i.e., pain, depression) [21, 23]. Research is emerging that involves a rigorous approach to CDS-Sx development. Cooley and colleagues adapted CPG for management of multiple co-occurring symptoms for use in an algorithm-based CDS-Sx that provided specific, tailored recommendations for symptom management at the point of care, and then tested the feasibility of implementation in a thoracic oncology outpatient setting [24, 25]. Researchers demonstrated a high level of complexity in the development and implementation of multiple symptom algorithms; in addition, almost 90% of symptom reports were delivered to clinicians before the patient’s visit, and clinicians adhered to the reports’ recommendations 57% of the time [26]. However, these algorithms were tested in one homogenous care delivery setting. The CDS-Sx system we developed extends previous interventions by delivering guideline-based management for nine symptoms that commonly occur among adults with cancer that are informed through the collection of symptom severity and symptom context information (symptom characteristics, remedies tried, comorbidities) directly from patients in near real time combined with EHR data to generate explicit, detailed, patient-tailored, actionable, pharmacologic and nonpharmacologic recommendations for oncology clinicians at the point of care.

**Fig. 1:**
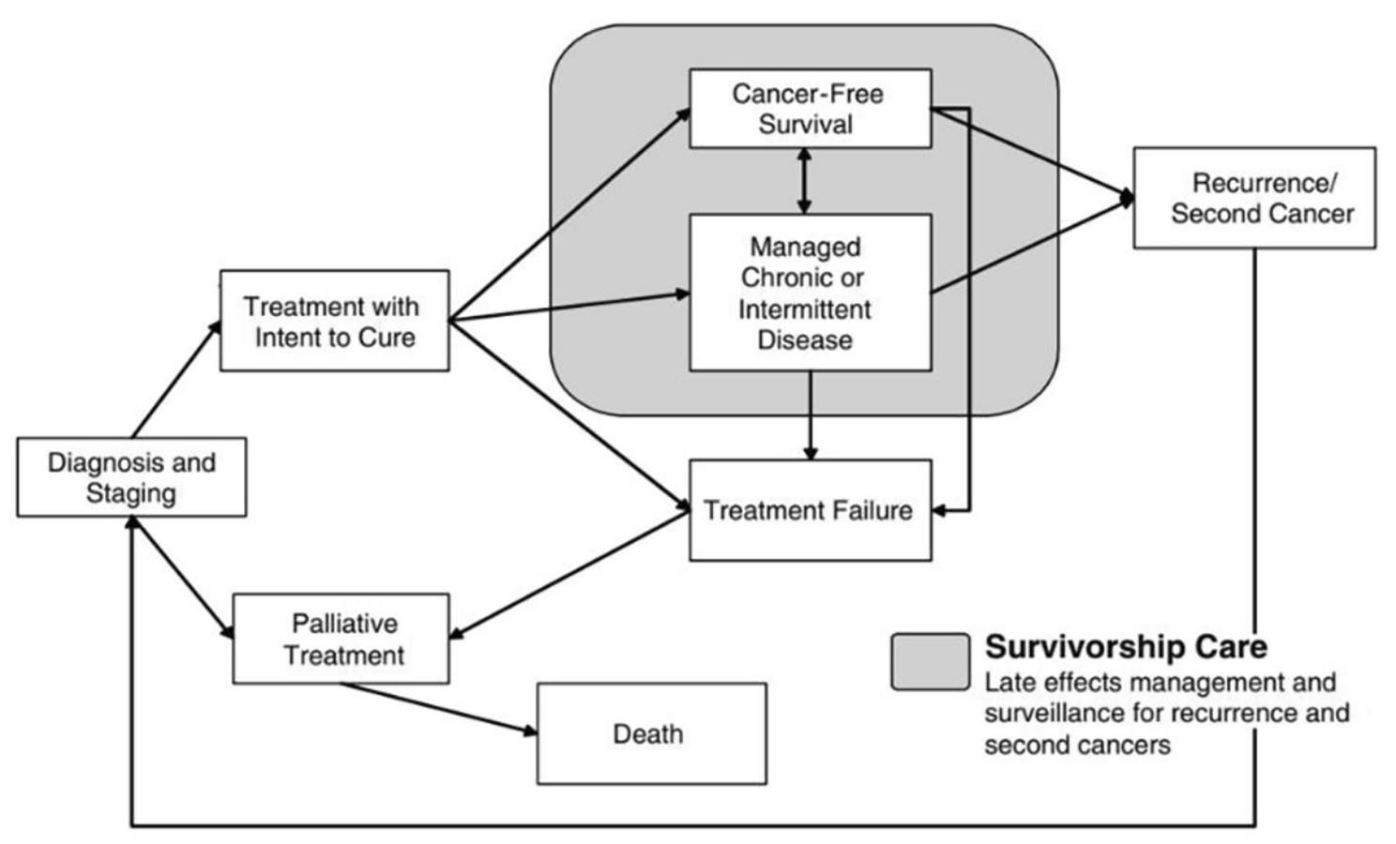
Cancer care trajectory. Used with permission of the National Academies Press, from Cancer Patient to Cancer Survivor: Lost in Transition, Maria Hewitt, Sheldon Greenfield, Ellen Stovall, Eds., 2005; permission conveyed through Copyright Clearance Center, Inc

Cancer symptom management is delivered across a variety of settings, each addressing different aspects of the cancer care continuum. The trajectory of cancer care, beginning with diagnosis and continuing to initial treatment, possible recurrence, and survivorship care is depicted in Fig. 1. CDS-Sx recommendations can be implemented at each stage of the care trajectory, and algorithms should be developed relevant to the needs of patients at each stage.

This study seeks to expand the literature by exploring whether algorithm-based CDS involving multiple symptoms can be applied across the cancer care continuum depicted in Fig. 1 and across cancer care delivery settings. This initiative was part of a Phase I Small Business Innovation Research (SBIR) contract funded by the National Cancer Institute (NCI) as part of the Cancer Moonshot Initiative focused on the development of new technologies to minimize cancer treatment side effects [27]. The overall approach requested by NCI was to create a CDS-Sx tool that collected symptom information directly from patients using validated patient-reported outcome (PRO) instruments and provided guidance to clinicians at the point of care by integrating evidence-based, individually tailored recommendations for cancer symptom management into the EHR. Treatment-aligned, symptom-focused patient educational materials were to be available to share with patients.

The objectives for this project were to develop and evaluate the usability of symptom management algorithms for two of the most common symptoms experienced by cancer patients, i.e., constipation and fatigue, which occurred in patients throughout their cancer trajectory, from active disease treated in comprehensive and community cancer centers to surveillance of cancer survivors in primary care practices. We excluded the end of life (EOL) phase of care from this project. The rationale for this exclusion was that EOL patients experience a unique array of symptoms and treatment considerations that differ greatly from those of adult cancer survivors or patients undergoing active treatment. For example, as adults approach EOL, oral medications may need to be replaced by rectal or subcutaneous medications. In addition, as EOL approaches, caregiver report often replaces patient report of symptoms and symptom severity. Problems due to organ system failure in these patients may create symptoms such as myoclonus and delirium that are uncommon in adults receiving cancer treatment in outpatient settings [28–30]. The unique integration of patient individualization and tailored recommendations using both patient and EHR data has not been utilized in prior CDS research.

## Methods

**Fig. 2:**
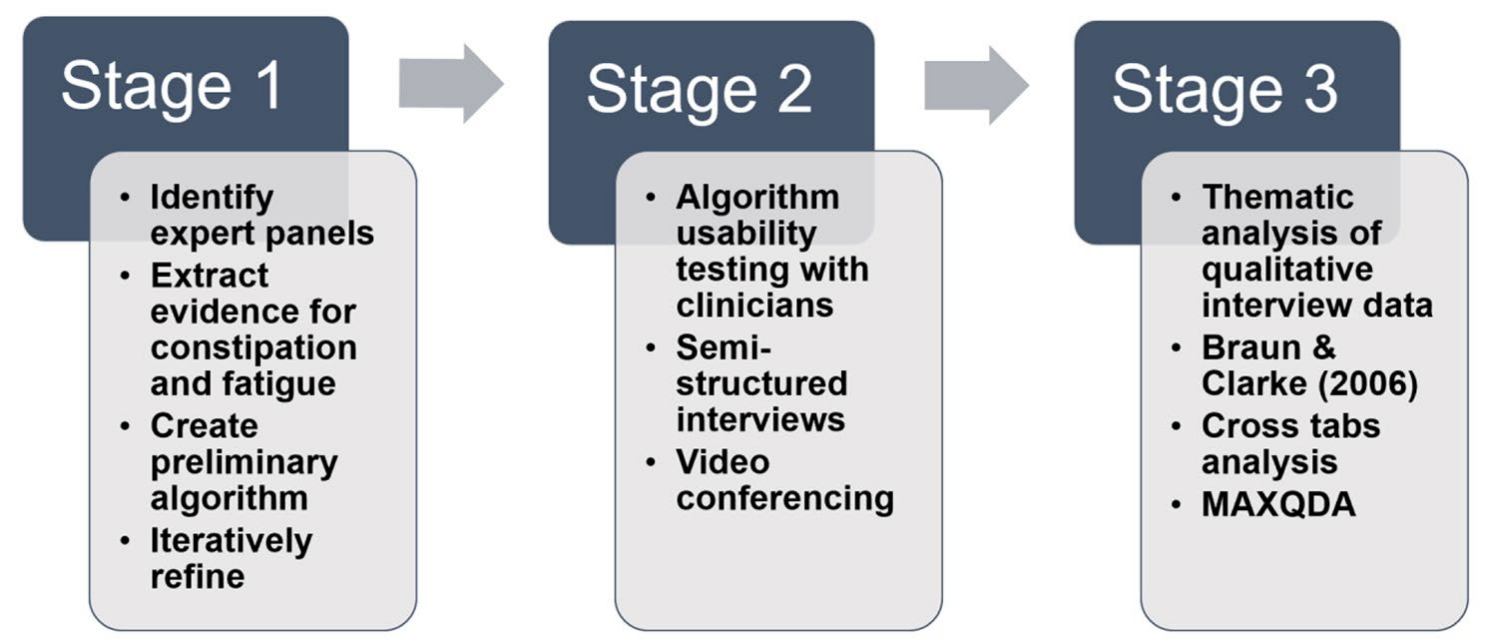
Process for developing and testing algorithm-based decision support for symptom management across the trajectory of cancer care

This study involved a 3-stage process for developing and testing algorithm-based CDS-Sx in patients from initial diagnosis, through survivorship, or in advanced stages of disease. These stages are depicted in Fig. 2 and include: (1) the algorithm development process, (2) algorithm usability testing with clinicians, and (3) thematic analysis of usability interview data [31].

### Stage 1: algorithm development process

A modified ADAPTE process, a replicable consensus-based methodology using expert panels, was used to develop algorithms guided by PRO measurements and extraction of data from the EHR. The algorithm development process has been described in further detail by Cooley and colleagues in a prior publication [25]. Overall, the algorithm development process for each symptom management team (constipation and fatigue) involved: (1) identification of expert panel team members, (2) extraction of evidence from national CPGs [8–11], (3) creation of a preliminary algorithm, (4) iterative refinement of the symptom management algorithm through panel discussions, (5) testing the algorithm with a panel of end users and (6) review of the end user feedback with the expert panel followed by iterative refinement as needed and then approval by the expert panel through consensus. Clinicians from multiple disciplines and clinical settings who manage patients with cancer symptoms were engaged to develop algorithms. Content experts were identified base on their specific expertise within Dana-Farber Cancer Institute and from the community affiliates and e-mail invitations were sent requesting their participation. All of the experts who were invited agreed to participate (19/19 for 100% response rate). Honoraria were provided as a token of appreciation for their time and expertise. A group size between two and fourteen participants is recommended as a sample size for assembling expert panels using a nominal group technique approach to achieve consensus [32]. Clinicians met face-to-face via videoconferencing, to align with the nominal group technique.

The research team conducted a review of the literature to identify current clinical practice guidelines for fatigue and constipation management from leading professional organizations and extracted information about the key intervention recommendations and levels of supporting evidence for each recommendation to create evidence tables [8–11]. Using these tables, clinical domain experts developed an initial algorithm “strawman” that was depicted using a Visio software program. Each expert panel member received the referenced tables of evidence and the “strawman algorithm” prior to the initial meeting to facilitate discussion. (Details of expert panel composition included in the Results Section.) The expert panels met three times to refine the algorithms. At a joint meeting, the two expert panels (i.e., on fatigue and constipation) reviewed the initial algorithms, identified elements that needed further refinement, and ultimately achieved consensus on algorithms to be tested by end users.

Each algorithm includes a comprehensive assessment element prior to any treatment recommendations. Causes for the symptoms are drawn from the evidence available in national CPGs. The NCCN does not specify cancer type as part of the assessment; rather, the problems that accompany the cancer that contribute to fatigue or constipation are included, such as anemia for fatigue, and hypercalcemia or concomitant opioid use for constipation. For each cause listed, the algorithm first suggests correction of that problem (e.g. anemia, hypercalcemia) when possible, before progressing to treatment suggestions that are tailored not to the type of cancer treatment, but to the individual patient data resulting from that treatment. For constipation, for example, a low platelet count and white blood count preclude rectal therapies; recent abdominal surgery precludes stimulant laxatives and metoclopramide. Further, the algorithm makes specific medication suggestions for treatment of constipation for patients taking opioids, and for those not taking opioids.

Clinician usability testing was initiated with paper versions of the algorithms to gather end-user feedback and ensure that the algorithms were acceptable across front-line clinical care providers in cancer care settings (nurse practitioners, physician assistants, and physicians). Other members of the allied health profession were not included in the algorithm testing since the recommendations suggested that clinicians initiate supportive care referrals to these professionals. These sessions were recorded and then analyzed for recurrent themes as described below. The algorithms were further refined by the research team based on clinician feedback and the revised versions were presented to the two expert panels in a second joint session, during which the algorithms received final approval.

### Stage 2: algorithm usability testing with clinicians

The algorithm usability testing phase of this study involved semi-structured formative qualitative interviews to vet the algorithms with practicing frontline clinicians. Usability testing was defined as reviewing a symptom algorithm in the context of a specific patient case study for feedback. Qualitative usability testing allows researchers to uncover problems and opportunities for improvement in their design [33]. The goal of sampling in usability testing is to identify target end-users who are able to provide robust feedback to improve the design and use of the product. Eligible participants were clinicians identified from a comprehensive cancer center, community hospitals, or primary care practices in New England, each a target practice type to reflect the environment in which the algorithms would be implemented. Inclusion criteria for participants specified clinicians who provided symptom management to patients with cancer in the past 6 months. Snowball sampling methods were used to identify and recruit clinicians known to study co-investigators or members of the expert panels. In usability testing, a sample size of five is recommended as the optimal sample size, which will detect 85% of user problems [34]. Three users per category is adequate when stratifying by different groups to examine the diversity of user behaviors [35]. Participants were English-speaking adults. IRB approval was obtained, and each clinician participant provided informed consent and permission to record the interview prior to data collection. Participants received a $100 gift card in remuneration for their involvement (DFCI protocol #18–662).

To illustrate how the algorithm would work in a clinical setting, clinicians who represented potential end-users of the symptom management algorithms were sent PDF files of an algorithm flow chart and patient-facing education materials for either constipation or fatigue and a relevant case study before an audio-recorded interview session. The case study described a clinical scenario that represented one of the complex paths through the algorithm that would result in generation of specific symptom management recommendations. The think aloud methodology was used to understand clinician perceptions and evaluation of the algorithms and how they could be integrated into clinical care [36, 37].

Clinician participants engaged in one 30-minute virtual semi-structured interview. The interviewer reviewed the algorithm, case study, and patient education materials with the clinician, and then elicited feedback on the content, specific recommendations, and how the algorithms could be integrated within the EHR to complement clinical workflow. The interview questions were structured with the following main topics: (1) Overall impressions of the algorithm, including sequence and flow, (2) Concerns or changes to algorithm recommendations, (3) Barriers to implementation, (4) Comfort level with use of algorithm in the clinical care setting, (5) Feedback on patient education materials, and (6) Symptom-specific feedback. Interview sessions with clinicians were conducted until researchers determined data saturation was achieved and no new themes emerged. The interview guide is included as a supplementary file.

### Stage 3a: thematic analysis of usability interview data

Each of the audio-recordings was transcribed. All identifying information was omitted from the transcripts. The transcripts were analyzed with MAXQDA, a qualitative data analysis software [38]. All transcripts were read in full to gain a general understanding of the data. Then, the data were analyzed across transcripts by two independent researchers using Braun and Clarke’s thematic analysis method [31]. This method involved: (1) Familiarizing yourself with the data, (2) Generating initial codes, (3) Searching for themes, (4) Reviewing themes, (5) Defining and naming themes, and (6) Producing the report [31]. This rigorous analysis method seeks to identify themes within data and provide an interpretation.

### Stage 3b: cross-tabs analysis for presence of themes across cancer care settings

A cross-tabs analysis was conducted in MAXQDA to assess the presence of themes by the varied cancer care delivery settings. This included primary care, community cancer centers, and comprehensive cancer centers.

## Results

The results of this study are presented in a stepwise approach to align with its three-stage process, (1) algorithm development, (2) algorithm usability testing, and (3) thematic analysis of usability interview data.

### Stage 1 results: algorithm development process

A total of 19 expert panel members participated in the development of algorithms for constipation and fatigue based on published CPG (Table 1). One clinician on each panel was also a cancer survivor.

**Table 1:** Expert panel member composition (*n* = 19)

Characteristic		Algorithm	
		Constipation (*n* = 8)	Fatigue (*n* = 11)
Training	Physician	4	5
	Nurse practitioner	1	2
	Pharmacist	1	1
	Nutritionist	1	1
	Nurse educator	1	0
	Psychologist	0	1
	Social worker	0	1
Practice setting	Comprehensive cancer center	5	7
	Community cancer center	2	3
	Primary care	1	1
Expertise*	Medical oncology	3	2
	Breast oncology	0	3
	Gastroenterology	1	0
	Palliative care	2	1
	Primary care	1	1
	Patient education	1	0
	Pharmacy	1	1
	Nutrition	1	1
	Integrative medicine	0	2
	Radiation oncology	0	1
	Social work	0	1

*Some members had multiple specialties

### Stage 2 results: algorithm usability testing with clinicians

A total of 17 clinicians participated in the algorithm usability testing for this study. The demographics for these usability participants are included in Table 2. Clinicians were from a variety of training and practice settings, and they either reviewed the constipation or fatigue symptom algorithm.

**Table 2:** Demographics of participants (*n* = 17)

Characteristic		Algorithm	
		Constipation (*n* = 8)	Fatigue (*n* = 9)
Training	Physician	1	4
	Nurse practitioner	3	3
	Physician assistant	4	2
Practice setting	Comprehensive cancer center	1	5
	Community cancer center	5	2
	Primary care	2	2
Age	20–29	0	1
	30–39	2	3
	40–49	4	5
	50–59	2	0
Gender	Female	8	7
	Male	0	2
Years of experience	Less than 5 years	2	2
	5–10 years	4	3
	10 + years	2	4

### Stage 3a results: thematic analysis of usability interview data

**Fig. 3:**
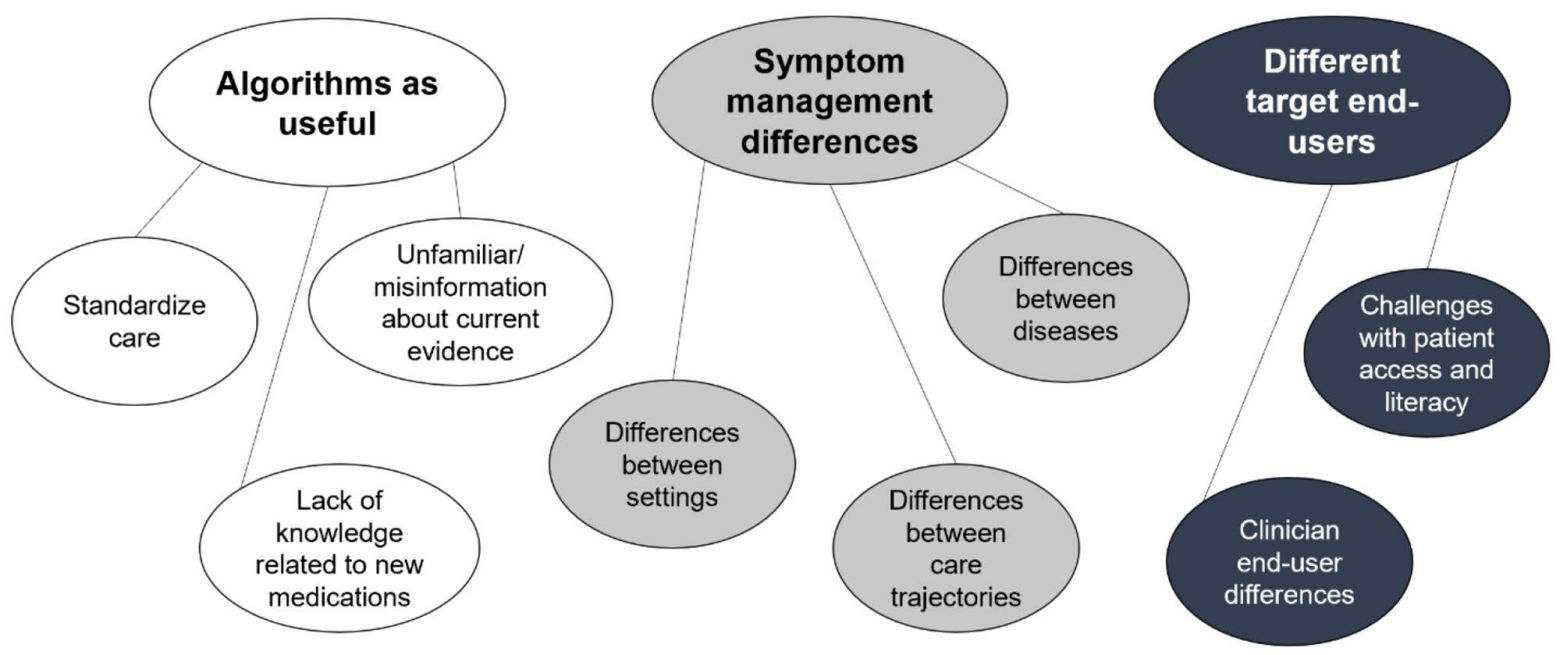
Depiction of themes and associated sub-themes

Using Braun and Clarke’s thematic analysis method [31], three major themes emerged from the data: (1) Algorithms as useful, (2) Symptom management differences, and (3) Different target end-users. Figure 3 depicts the identified themes and associated sub-themes. The main themes are represented by the top tags in the figure and the sub-themes are connected by lines below each main theme.

A total of 114 codes were extracted from the transcripts. Table 3 depicts the frequency of the codes and overall themes from the MAXQDA software to demonstrate how the most prevalent themes were identified. The number of participants who contributed to each code is also depicted. In the following paragraphs, the included quotes were chosen as representatives of each theme or sub-theme.

**Table 3:** Frequency and participant report of codes

Theme	Code	Frequency of code	Number of participants reporting code
*Algorithms as useful*			
	Algorithms as useful	31	17
	Standardize care	15	8
	Unfamiliar/ misinformation about current evidence	6	5
	Lack of knowledge related to new medications	15	10
*Symptom management differences*			
	Differences between settings	22	5
	Differences between care trajectories	8	1
	Differences between diseases	2	2
*Different target end-users*			
	Clinician end-user differences	4	3
	Challenges with patient access and literacy	14	8

### Theme 1: algorithms as useful

The most prominent theme that emerged from the interview data was the perceived usefulness of CDS-Sx algorithms. As participants considered the implementation of the algorithm-based CDS in their setting, comments included: “*This is desperately needed*,” and “*This would be extremely helpful*.” The algorithms were perceived as thorough and efficacious by the participants.

*I think overall, it’s very comprehensive, it’s easy to follow, it’s a good thing to institute.*

Participants also considered how helpful implementation would be to their current practice.

*Perhaps if we followed the algorithm precisely maybe we’ll have more success.*

#### Subtheme: standardize care

Participants expressed appreciation for the specific recommendations and comprehensive nature of the guidance. One participant who reviewed the constipation algorithm stated:

*I think it’s a great idea for having everybody on the same page because you will see different providers recommend different things.*

Participants acknowledged that differences in symptom management are common and algorithm-focused care would eliminate these inconsistencies.

*I look forward to being able to use this because I think it’s very practical and will be useful for nurses and providers to give a standardized response.*

Additionally, clinicians reviewed the patient education materials that were developed in conjunction with the symptom algorithms.

*It’s just so helpful to have a standardized plan to give patients and to have the resources to give them education materials on their particular problem.*

#### Subtheme: unfamiliar/misinformation about current evidence

While some participant comments highlighted the value of care standardization, other statements reflected misinformation regarding current evidence. Each algorithm included recommendations from national CPG, but some participants made statements refuting guidance.

*It is not realistic to send [patients] to exercise programs. I [discuss] energy preservation and tell them it’s OK to sleep in the middle of the day.*

Variability in symptom management practices was evident in some interviews, with participants explaining why they would not follow certain recommendations. For example, in response to the algorithm’s omission of docusate as a first-line treatment for constipation (based upon randomized trial data that it is ineffective [39]), one participant stated:

*Colace is a stool softener, not a laxative. So, it doesn’t increase motility… having a stool softener upfront is not a bad idea.*

In addition to some participants disagreeing with recommendations, others recognized that they did not regularly follow the current evidence-based guidance for constipation.

*I don’t know that I necessarily consistently maximize the dose before moving on to the next step, so I like having the reminder to utilize each medication to its maximum amount and then move on.*

#### Subtheme: lack of knowledge related to new medications

Participants expressed a lack of knowledge related to new medications found in the current evidence (e.g., lubiprostone, linaclotide, naldemedine), particularly as they reviewed the constipation algorithm. One participant stated:

*I am not familiar; I’ve never used some of these medications.*

Some commented that they would be willing to prescribe or recommend medications that were new to their practice, pending their own review of the nuances of the medication:

*They’re definitely something that I would need to look up if I ever needed to use them.*

### Theme 2: symptom management differences

The second theme that emerged from the interview data was “Symptom management differences,” reflected in differences between settings, care trajectories, and diseases. Participants were recruited from comprehensive cancer care, community cancer care and primary care, reflecting the varying settings for symptom management for patients with cancer.

#### Subtheme: differences between settings

Primary care clinicians discussed the challenges they would have in implementing symptom algorithms in their setting. When considering the recommendations in the fatigue algorithm, one participant stated:

*From a primary care perspective, in general we don’t use a lot of Ritalin in this context… I would certainly be willing to do it, especially if a guideline like this was helping me with it.*

Participants expressed how their care setting influences how they view the symptom management of patients with cancer. Specifically, primary care clinicians discussed how they focus on managing constipation symptoms with dietary changes instead of medication prescriptions.

*I pay more attention in family medicine about what diet is, so I start at the very top, because you see what they are eating and their fluid intake, because yes, they are having constipation, but they just started the Keto diet two weeks ago.*

#### Subtheme: differences between care trajectories

The sub-theme of “Differences between care trajectories” emerged as participants reflected on the usability of algorithms across the care continuum.

*Breaking it down that way, between active treatment and advanced disease, it covers the major grouping of people we see…there are different specifics in each of those different pathways. The survivorship one didn’t feel as familiar, as I tend to not have a lot of interaction with people with no incidence of disease and off treatment.*

#### Subtheme: differences between diseases

This sub-theme included a discussion of algorithm implementation across different cancer diagnoses. One participant noted the use of patient reported outcome measurements (PROMs) and that symptom questions could be tailored to disease-types:

*I would say every patient at every visit gets to fill out a PROMs. With specific things that fit to them, for example someone with prostate isn’t going to be talking about esophageal cancer symptoms.*

Participants also commented on their disease-specific clinics and their concern with the algorithm’s ability to address the specific issues of their patient population.

*My only concerns are specific applicability to our clinic, so I work in leukemia, all of our patients are pancytopenic. So, you know these are all things that contribute to their fatigue but a lot of times their fatigue is primarily secondary to being anemic or neutropenic.*

In all, participants considered implementation of the CDS-Sx algorithms in their care settings but noted certain nuances in disease-specific clinics.

### Theme 3: different target end-users

The final theme that emerged in the data involved the target end-users for the algorithms. Many of these codes stemmed from participant reflection on the algorithm and the appropriate target end-users.

#### Subtheme: clinician end-user differences

In the interviews, participants reflected on the implementation of the algorithms within their clinic settings. A recurrent comment was the notion that there were certain clinician end-users that could benefit more from the CDS-Sx algorithm than others.

*This is the sort of thing that our providers are looking for, especially those advanced practice providers, where they are open to new ideas where the physicians might not be.*

The concept that there were clinicians who would be more receptive to algorithm-based symptom management also extended to the level of experience and complexity of patient populations in an end-user’s setting.

*I think the algorithm would be more helpful for new people versus someone who has been practicing for 25 years, but also for more complicated patients.*

#### Subtheme: challenges with patient access and literacy

Participant comments involving patients included a reflection on patient access and health literacy to consider when implementing CDS-Sx. The communities where participants provided care included areas with diverse populations, *“multiple languages,”* and patients with limited language proficiency.

*I mean certainly some of our patients who are tech-savvy will be able to access the system but a lot of our patients either don’t speak English or don’t read… or have low health literacy.*

Community care center participants discussed the population differences present across their settings that may impact patient use of CDS-Sx.

*In our population, language barrier is a considerable barrier. We do have a patient portal and we have few patients who will actually use that, because they do better more one-on-one. I would say in our population, there would probably be few that would do that on their own without prodding from the staff.*

### Stage 3b results: cross-tabs analysis for presence of themes across cancer care settings

Table 4 depicts the presence of themes across the three care settings. The cross-tabs analysis uncovered distinct patterns across the sub-themes in Theme 2, “Symptom management differences.” The sub-theme of “Differences between settings” was reflected in primary care and community cancer settings. In contrast, every reference code for the sub-themes “Differences between care trajectories” and “Differences between diseases” emerged from comprehensive cancer settings. Additionally, the Theme 1 sub-theme of “Standardize care” was not present in primary care settings.

**Table 4:** Presence of themes by type of cancer care setting

		Comprehensive cancer center	Community cancer center	Primary care
Algorithms as useful		Yes	Yes	Yes
	Standardize care	Yes	Yes	No
	Unfamiliar/ misinformation about current evidence	Yes	Yes	Yes
	Lack of knowledge related to new medications	Yes	Yes	Yes
Symptom management differences				
	Differences between settings	No	No	Yes
	Differences between care trajectories	Yes	No	No
	Differences between diseases	Yes	No	No
Different target end-users				
	Clinician end-user differences	Yes	Yes	Yes
	Challenges with patient access and literacy	Yes	Yes	Yes

## Discussion

We developed and tested algorithms that will provide individualized recommendations to enhance CPG-based symptom management for cancer-related fatigue and constipation at the point of care. Health information technologies are evolving quickly and are being used to decrease fragmentation of care and improve the quality of cancer care delivery [40]. The deployment of health information technology alone is not enough to improve the quality of cancer care delivery. It is essential to involve the target end-users in the development of these technologies so that the tools developed will be easy-to-use, intuitive, and useful [40, 41].

The results from our usability testing identified that clinicians perceived that the algorithms were useful and provided up-to-date information about new treatments. Participants highlighted the perceived utility in comments in which they envisioned themselves using the algorithms in their current practice. Additionally, the comprehensive nature of the algorithms supported their usefulness in care settings where they were seen as a pathway to successful symptom management. Our study expanded previous work in cancer patients, in which the perceived usability of algorithm-based CDS was demonstrated, acceptability was found to be favorable, and participants provided many suggestions for improvement based on their current practice [42].

This study found that some clinicians had misconceptions about best practices, especially related to cancer-related fatigue. Even though there are multiple CPGs for the management of cancer-related fatigue [9, 11], implementation of these CPGs into routine cancer care remains limited. Jones and colleagues interviewed clinicians about their experience and opinions about the use of cancer-related fatigue CPGs and the underlying causes for treatment gaps [43]. As in our study, clinicians lacked knowledge about the existence of and/or content for appropriate cancer-related fatigue interventions.

Previous studies examining the use of CDS in oncology have focused on a wide array of applications including reducing medication errors through the use of computerized chemotherapy order entry, enhancing adherence to cancer treatment guidelines through the use of clinical pathways and improved identification of eligible patients for clinical trials [44, 45]. Fewer studies have focused on improving patient-reported outcomes. Most of the CDS systems focused on improving symptom management have used alert systems to notify clinicians of increased symptom severity or providing patients with educational information to promote symptom management [18, 19]. Only one previous study examined the use of CDS to promote adherence to guideline-concordant care for multiple symptoms among adults with advanced lung cancer [46]. Clinicians were randomized to receive CDS that provided individually tailored recommendations to improve the management of multiple symptoms (pain, fatigue, dyspnea, depression, anxiety) or to a usual care group. Results of the study indicated that patients assigned to the CDS treatment group were more likely to receive sustained-release opioids for constant pain, adjuvant medications for neuropathic pain, opioids for dyspnea, and stimulants for fatigue as compared with the usual care group [46]. Our current study advances the previous work by updating and expanding the cancer symptom management algorithms and by integrating the CDS-Sx system into the EHR workflow using Health Level 7 standards to make the system interoperable and scalable. It is important to note that guidelines may change over time as new evidence becomes available. It is imperative that CDS systems have a process in place for a regular review and updating of the algorithms in order to provide guideline-concordant care.

Differences in the perception of care standardization emerged between primary care and cancer care settings, as primary care clinicians did not discuss standardization of care. Their comments highlighted differences between the algorithm recommendations and their current approach to symptom management, since the etiology and initial treatment for fatigue and/or constipation often differs among patients receiving active treatment compared with survivors receiving care in a primary care setting. Identification and treatment of physical and psychosocial symptoms are only one aspect of quality survivorship care. Nekhlyudov et al. identified key domains of quality care that need to be addressed for cancer survivors, which included: prevention and surveillance for new cancers and/or recurrence, management of co-occurring chronic medical conditions and health promotion/disease prevention [47]. Further development of CDS-Sx systems that facilitate care coordination among oncology, primary care and specialty care settings is needed [48]. However, these systems must address the unique needs of cancer survivors who are post-treatment and address their long-term and chronic needs as they transition to primary care settings [49].

Differences in symptom management practices also emerged between comprehensive and community cancer settings. The clinicians in comprehensive cancer settings identified the need for disease specific measurement and recommendations since they often focused on providing care to patients with a specific type of cancer. Moreover, some clinicians identified that they were not familiar with the post-treatment trajectory of care since they focused on providing care to those undergoing active cancer treatment. Clinicians in community-based settings did not indicate the need for disease specific measures since they see a heterogeneous group of cancer patients across the trajectory of care. This difference is an important finding that has relevance for future development of CDS-Sx systems. Kaufmann and Rocque recently noted that the next step in moving PRO measurement systems forward is to tailor PROs for individuals based on individual, disease and treatment characteristics and then to use that information as an intervention when patients pass a certain threshold for symptom severity [50]. Standardized PRO measurement is available to gather information about core symptoms that are common across cancers as well as disease specific symptoms to enable more precise measurement for relevant symptoms [4, 51, 52]. Thus, further development and testing of algorithm-based CDS systems using symptom pathways that can be individually tailored can fill this gap in care.

Advanced practice practitioners and clinicians with less experience in the clinical setting were identified as being more open to the use of algorithm-based CDS to manage cancer symptoms. Our findings are similar to other studies that found that younger clinicians and those with less work experience were more likely to use algorithm-based CPG [53]. Involvement of the target group in the development, testing and deployment of the algorithm-based CDS is an essential first step in enhancing successful implementation [54]. The findings from this study suggest that advanced practice practitioners may be a good target for future implementation efforts. This finding is consistent with previous studies that identified that advanced practice practitioners, especially nurses, are in a key position to manage symptoms during and after cancer treatment. Innovative models of nurse-managed symptom management programs have resulted in improved outcomes including adherence to CPG-based care, decreased symptom burden, decreased hospitalizations, and increased completion of intensive chemotherapy regimens [55–58].

Clinicians identified that some patients would not be able to complete the electronic patient-reported outcome questionnaires that are necessary to elicit the CDS-Sx recommendations due to not having access to technology and/or limited proficiency in the English language. In this regard, the implementation of PRO systems, including CDS-Sx, has the potential to exacerbate health disparities. Recent evidence suggests that a digital divide related to health-related Internet usage persists among cancer survivors, especially among those that are older, ethnic minorities, less educated, and residing in rural communities [59]. It is critical that future research focus on ways to enhance equity through the translation of PRO questionnaires, validation in underserved populations, and implementation strategies to increase uptake of CDS-Sx systems in diverse cancer care settings [50].

### Strengths and limitations

A limitation of this project included the overrepresentation of participants from a New England-based comprehensive cancer center and community cancer centers in both the expert panels and the usability evaluation study. Most usability participants were female, ages 30–49, limiting the generalizability of findings. This study focused on only two common cancer-related symptoms. However, the goal for this project includes obtaining additional SBIR funding to expand algorithm development to encompass six additional symptoms. We did not use a quantitative questionnaire for the usability assessment since participants reviewed electronic PDF or paper versions of the algorithms. A case study was used to illustrate individualized recommendations to enable clinicians to evaluate the appropriateness of the recommendations and provide rich qualitative data, which would not be possible with a quantitative questionnaire. The algorithm development and usability process that we used was a refinement process that engaged end-users in the co-design of the algorithms [60, 61]. This step was necessary before computing the algorithms to ensure that the algorithm content is appropriate for varied settings and clinician end users. A quantitative assessment questionnaire is appropriate when assessing an electronic or EHR-embedded tool [62]. Future research will include usability testing of electronic versions of the algorithms integrated into the EHR, and testing the effectiveness of the CDS-Sx system in multiple cancer centers.

## Conclusion

While CDS-Sx can be helpful with promoting evidence-based cancer symptom management, the CDS systems providing care recommendations will need to be specifically developed to fit the patient characteristics and context. This study identified that one set of algorithms will not be applicable throughout the entire cancer trajectory. Unique CDS-Sx will be needed for patients who are cancer survivors being followed in primary care settings. The CDS developed in this study is most applicable to patients being treated in comprehensive and community-based cancer settings who are suffering from symptoms related to their cancer after completion of treatment or during active treatment.

### Electronic supplementary material

Below is the link to the electronic supplementary material.

Supplementary Material 1

## Data Availability

The datasets used and analyzed during the current study are available from the corresponding author on reasonable request.
