# Supplementary material for: A qualitative analysis of algorithm-based decision support usability testing for symptom management across the trajectory of cancer care: one size does not fit all
Source: BMC Med Inform Decis Mak. 2024 Mar 5;24:63. doi: 10.1186/s12911-024-02466-7 (PMC10913367; doi:10.1186/s12911-024-02466-7)
Supplement: Supplementary file 1 — Supplementary Material 1 [file 12911_2024_2466_MOESM1_ESM.pdf]

CLINICIAN INTERVIEW GUIDE

Hello, thank you so much for participating in this session. Before we start, I want to confirm that you have reviewed and had the opportunity to ask about the purpose and procedures of the research study and consent to participate and have this session audio recorded. Is that all correct?

> <u>If no to any</u>: Stop and discuss the study, elements of consent, answer questions, and obtain consent to participate and be recorded prior to continuing
>
> <u>If yes</u>: Thank you, I am recording this session

We've arranged time to talk with you today and get your input on an algorithm for [constipation]/[fatigue] in patients with cancer receiving care across the disease trajectory, for example patients who are undergoing active treatment, those who are survivors, or those entering end of life. I'll also ask about a set of smart phrases that can be used to document interventions in the electronic health record.

I won't take more than about 30 minutes of your time, so let's get started.

First, for about 5 minutes, I'll tell you how the algorithm was developed and walk you through the algorithm's decision nodes and recommendations. After that, I'll ask for your overall feedback first, then drill down with specific questions. Okay?

The algorithm was developed by an expert panel consisting of cancer survivors and those with expertise in palliative care, oncology, gastroenterology, primary care, pharmacy, nursing, diet, behavioral science, informatics, quality improvement and patient education. The algorithm and smart phrases that we will show you focus on [constipation]/[fatigue] during active treatment, in primary care or the end of life. The suggestions were developed based on the literature, clinical guidelines and expert opinion.

[Show the algorithm and walk through the decision nodes]

Now that you've learned about the algorithm, the questions we have for you are:

1. What are your overall impressions of the content of the algorithm?
2. What do you think about the sequence and logical flow of the paths through the algorithm?

3. [Review each main node or node-set and solicit feedback]: Is there anything you would change here or concerns you would have? What do you think about the recommendations?
4. Overall, would you add/remove anything that is being suggested, or is there anything you would like to see different?
5. Could you talk about any barriers you would envision to implementing use of the algorithm in the setting where you work?
6. Would you personally be open to using this algorithm in care for patients in your setting? Would you feel comfortable having your patient receive care using this algorithm?
7. Do you currently use templates or smart phrases for charting, and is it clear how these smart phrases would be used with the algorithm?
8. Is there anything else that you think would make it easier for you implement use of the algorithm in your setting?

OK, before we finish up and I ask you to respond to a short demographic questionnaire, is there any other general feedback you would like to let our team know?

[Administer Clinician Participant Demographic Questionnaire]

Finally, is there any other clinician you would recommend we try to interview to get their feedback on this algorithm or on another one for cancer-related [fatigue]/[constipation]? You can also let us know later if you think of someone.

I want to thank you so much for your time today for your contributions to this research study. As a token of our team's thanks, I will be emailing you a gift card from Amazon.com. Let me just confirm your email address: [Confirm and read back].

Thank you again. Have a good day!
